# A size-structured matrix model to simulate dynamics of marine community size spectrum

**DOI:** 10.1371/journal.pone.0198415

**Published:** 2018-06-07

**Authors:** Shujuan Xia, Takashi Yamakawa

**Affiliations:** Department of Aquatic Bioscience, Graduate School of Agricultural and Life Sciences, The University of Tokyo, Tokyo, Japan; Swedish University of Agricultural Sciences and Swedish Institute for the Marine Environment, University of Gothenburg, SWEDEN

## Abstract

Several types of size-based models have been developed to model the size spectra of marine communities, in which abundance scales strongly with body size, using continuous differential equations. In this study, we develop a size-structured matrix model, which can be seen as a discretization of the Mckendrick-von Foerster equation, to simulate the dynamics of marine communities. The developed model uses a series of simple body size power functions to describe the basic processes of predator–prey interactions, reproduction, metabolism, and non-predation mortality based on the principle of mass balance. Linear size spectra with slopes of approximately –1 are successfully reproduced by this model. Several examples of numerical simulations are provided to demonstrate the model’s behavior. Size spectra with cut-offs and/or waves are also found for certain parameter values. The matrix model is flexible and can be freely manipulated by users to answer different questions and is executed over a shorter numerical calculation running time than continuous models.

## Introduction

Since the pioneering work by Sheldon *et al*. [1], the marine community size spectrum, *i*.*e*., the distribution of abundance or biomass against individuals’ body size on a log–log scale, has attracted significant interest from the scientific community. Classifying individuals over the size range from bacteria to whales into different size groups regardless of taxonomy results in a linear size spectrum with a slope of approximately –1 in unexploited communities [1, 2]. The slopes and intercepts of these size spectra have been proposed as useful indicators in revealing the nature of ecosystems and the effects of anthropogenic perturbation on the structure of marine communities [3].

Several types of size-based models have been developed to reproduce linear size spectra, explain their remarkable regularities, and explore the related ecosystem dynamics [4–8]. Such models are also widely used to investigate the impacts of environmental changes [9, 10] and fishing [11–13] on marine communities. Such models have been used to suggest that different types of behaviors can theoretically emerge at various size spectra. Using a jump-growth model in which predation is represented as a Markov process, Datta *et al*. [14] demonstrated that the size spectrum can switch from a linear to a wave-like shape when predators feed over a relatively narrow size range of prey.

In these studies, the balance between mortality, growth, reproduction, metabolism, *etc*. at all body sizes is formalized by using the Mckendrick-von Foerster partial differential equation [15]. Partial differential equation models (PDE models) enable the incorporation of detailed processes with various parametric functions. Although the R package “*mizer*” developed by Scott *et al*. [8] has been widely used for implementing size-based models, it may not always be easy or convenient for users to couple processes such as environmental stochasticity to PDE models in obtaining fits to the real situations of specific marine communities [16]. Matrix models can be more easily manipulated than PDE models in modeling such processes because they use simple discrete states [16]. Matrix algorithms can also be executed within shorter running times than continuous models with numerous loop iterations. Because real data are always recorded in discrete form, matrix models can be calibrated and validated with real data points.

In marine ecosystems, body size correlates with various biological and physiological processes; for example, the vital rates at the individual level, including encounter rate, swimming speed, reproduction rate, metabolic rate, and natural mortality rate, all ubiquitously scale allometrically with body size [7, 17–20]. Such processes are considered to be the main drivers that affect the dynamics of abundance and, because they can be described by simple power law functions of body size, they can be easily incorporated into a matrix model. Models such as the Leslie matrix model [21] have been used in population ecology to predict population growth and the age composition of the population. These models can provide clues for the construction of a size-structured matrix model for marine communities. Motivated by this background, the main goal of this study was to develop a simple and widely applicable size-structured matrix model that can easily be modified and extended to suit different applications in flexibly investigating community- and population-level dynamics.

In the following, we first present matrices corresponding to the basic processes of community dynamics to construct a unified matrix model. We then provide several numerical simulation examples in which the values of the model parameters are varied separately in a range around their reference values. The effects of fishing on stationary size spectra are then predicted under various fishing scenarios. Finally, we discuss the model structure and behavior of the observed size spectra and provide some modifications of the model for future applications.

## Materials and methods

### Size-structured community dynamics

Consider a size-structured community (*e*.*g*., marine community) in which larger organisms prey on smaller organisms (in this paper, we do not discriminate the identities of species). We begin construction of the matrix model by discretizing individuals with different body masses into *k* equally-spaced logarithmic size classes. The community structure is represented as a column vector **n**(*t*) = (*n*_1_(*t*),*n*_2_(*t*),…,*n*_*k*_(*t*))^T^, where the superscript T denotes vector or matrix transposition. Each value in the vector represents the number of individuals per unit volume measured in ind./m^-3^ within the class at time *t* (for brevity, in the following we omit writing (*t*) for the individual size classes). Let **m** = (*m*_1_,*m*_2_,…,*m*_*k*_)^T^ be the column vector describing the body masses of individuals in each size class. The body mass axis is discretized using a mass ratio of successive size classes *δ*: *m*_*i*+1_ = *m*_*i*_*δ*, where *i* = 1,2,…,*k*. In our model, the community is divided into two groups: the background resources’ group, which has body masses *m*_1_ and produces the only input to the community; and the consumers’ group, which has body masses belonging to [*m*_2_,*m*_*k*_].

**Fig 1:**
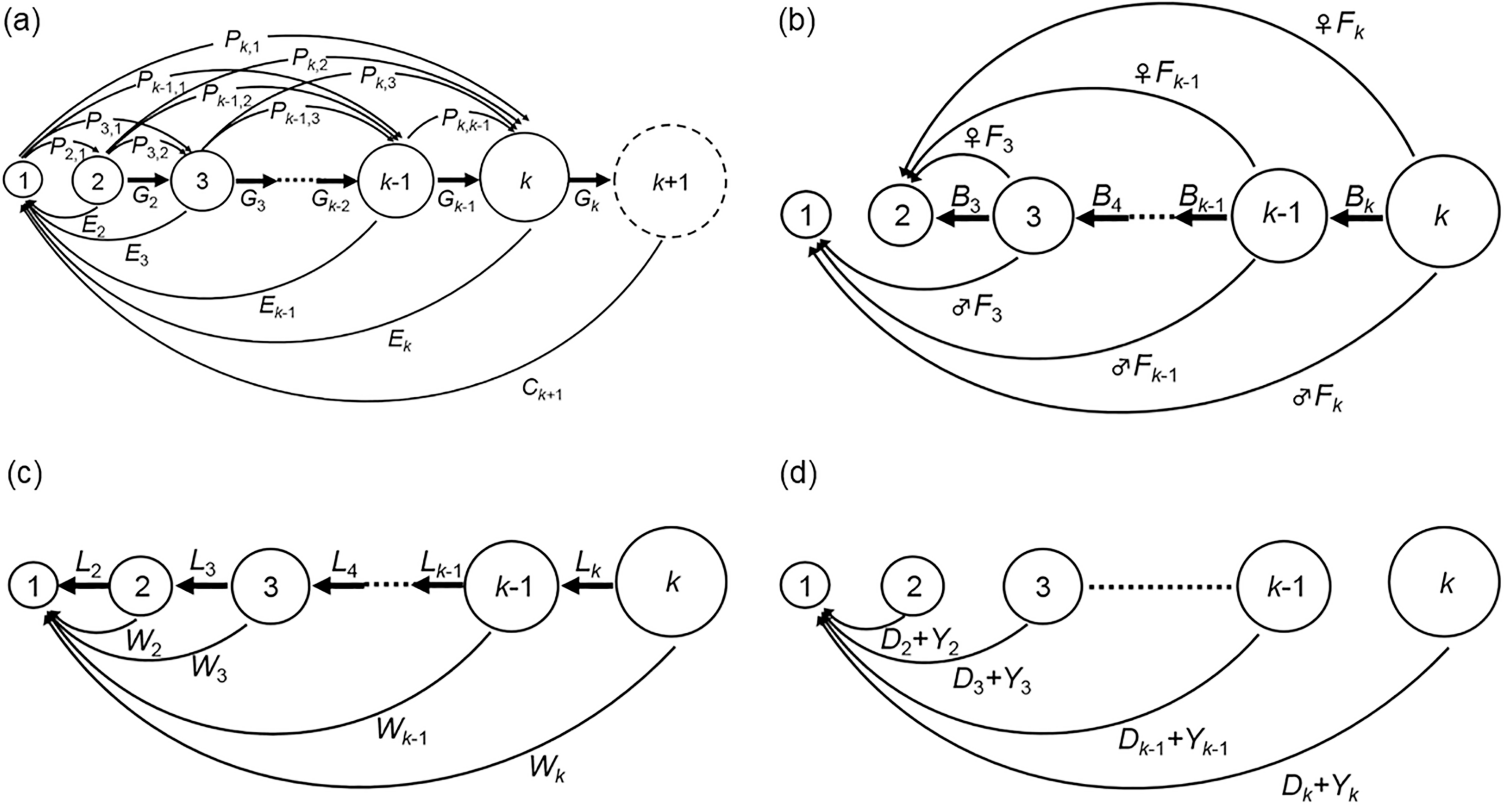
Graphic representation of the size-structured matrix model. Nodes represent size classes; larger circles depict larger body mass. Background resources are assumed to occupy only the first size class and consumers to range from the second to the *k*th size class. Thin arrows indicate flow of matter and energy expressed in mass (*E*, *C*, *F*, *W*) or number of individuals (*P*, *D*, *Y*) between size classes through each process per unit time Δ*t*, while thick arrows indicate transfer of individuals (*G*, *B*, *L*) between size classes owing to increase or decrease in body mass. Individuals can grow or move down no more than a single size class per unit time Δ*t*. (a) Predator–prey interactions: in a predation process, several individuals *P*_*i*,*j*_ in prey size class *j* are eaten by individuals in predator size class *i*. Undigested materials *E*_*i*_ are egested and recycled to resources in size class 1. After feeding, a certain fraction of individuals *G*_*i*_ in predator size class *i* grow to the next size class *i+*1 owing to mass increase. For individuals in the largest size class *k* in this community, we assume that no predation occurs (*i*.*e*., *P*_*i*,*k*_ is zero for all *i*). Instead, we assume that all individuals growing from size class *k* into an imaginary larger and empty size class *k*+1 out of this community die and the carrion *C*_*k*+1_ is recycled to resources. (b) Reproduction: the first size class of the consumers’ group (*i*.*e*., size class 2) is represented as the starting point of consumers as eggs or larvae. Males and females reproduce at the same reproduction rate *F*_*i*_, producing eggs into size class 2 (females) and excretive matters (semen) recycled to resources (males). As a consequence, a certain fraction of individuals *B*_*i*_ in parent size class *i* decrease their body mass and are transferred to the next-lower size class *i*–1. (c) Metabolism: individuals within size classes 2 to *k* produce metabolites that are recycled to resources at a rate *W*_*i*_, with a certain fraction *L*_*i*_ transferred into the next-lower size class owing to loss in body mass. (d) Non-predation and fishing mortality: several individuals *D*_*i*_ die owing to natural (non-predation) mortality; meanwhile, their carrion is recycled to resources. Fishing removes some individuals *Y*_*i*_ from each size class *i*. These catches are consumed by humans and eventually reintegrated into the recycling process and back into the ecosystem as nutrients to resources. In our model, they are recycled in size class 1 while maintaining the mass balance.

The model, presented graphically in Fig 1, shows four processes that affect the number of individuals in each size class per unit time Δ*t*. The dynamics of community size structure can be described in terms of these basic processes of predator–prey interaction, reproduction, metabolism, and non-predation mortality. As described below, the model presented here relies on empirical knowledge (*e*.*g*., allometric relationships) between body mass (*m*) and the rates or quantities of organism physiological phenomena (*y*) according to simple power functions *y* = *am*^*b*^, where *a* is a coefficient and *b* is a scaling exponent. Our model is designed based on a mass balance in the community (*i*.*e*., no biomass is wasted). Because materials such as dead animals, fecal pellets, and metabolites from consumers constitute the assimilable inorganic and organic nutrients needed for phytoplankton growth, we assume that all egested materials (*i*.*e*., undigested materials, metabolites, carrion, *etc*.) are assimilated to form new resources through microbial degradation such that a simple modeling of the dynamics of resources is possible. To maintain the mass balance, an imaginary size class *k*+1 is introduced to remove the increased biomass in size class *k* induced by predation.

Assuming that the basic processes in Fig 1 are independent of each other, the column vector **n**(*t*+Δ*t*) representing the community structure at time *t*+Δ*t* is given by

$$n(t+\Delta t)=n(t)+\Delta n^{P}+\Delta n^{R}+\Delta n^{Q}+\Delta n^{U}$$ (1)

where Δ**n**^*P*^, Δ**n**^*R*^, Δ**n**^*Q*^, and Δ**n**^*U*^ are column vectors ($\Delta n^{P}={\left(\Delta n_{1}^{P},\Delta n_{2}^{P},\ldots ,\Delta n_{k}^{P}\right)}^{T}$, $\Delta n^{R}={\left(\Delta n_{1}^{R},\Delta n_{2}^{R},\ldots ,\Delta n_{k}^{R}\right)}^{T}$, $\Delta n^{Q}={\left(\Delta n_{1}^{Q},\Delta n_{2}^{Q},\ldots ,\Delta n_{k}^{Q}\right)}^{T}$, and $\Delta n^{U}={\left(\Delta n_{1}^{U},\Delta n_{2}^{U},\ldots ,\Delta n_{k}^{U}\right)}^{T}$) describing shifts in the number of individuals within each size class as a result of predator–prey interactions (*P*), reproduction (*R*), metabolism (*Q*), and non-predation, and fishing mortality (*U*), respectively.

### The matrix model

#### Predator–prey interactions

In each predation event, we assume that predators have a preferred predator–prey mass ratio (PPMR) based on the rule “bigger ones eat smaller ones” [22]. A size selectivity matrix **S** by a predator in size class *i* on a prey in size class *j* composed of size preferences *φ*_*i*,*j*_ (*i*>*j*) and zeros is described as follows:

$$S=\left(\begin{array}{ccccc} 0 & 0 & 0 & \cdots & 0\\ \varphi_{2,1} & 0 & 0 & \cdots & 0\\ \varphi_{3,1} & \varphi_{3,2} & 0 & \cdots & 0\\ \vdots & \vdots & \ddots & \ddots & \vdots \\ \varphi_{k,1} & \varphi_{k,2} & \varphi_{k,3} & \cdots & 0 \end{array}\right)$$ (2)

where *φ*_*i*,*j*_ is given by a function that follows a lognormal distribution on the ratio of body masses of predator and prey, *m*_*i*_ and *m*_*j*_, respectively [22]. Although resources are only placed at the first size class [*m*_1_] in our model, we assume that they also extend to an imaginary size range [*m*_−*s*_,*m*_0_] (which is divided into logarithmically equally spaced *s*+1 size classes, and *m*_0_<*m*_1_) in calculating *φ*_*i*,*j*_ to ensure that small consumers can feed on preferred prey smaller than *m*_1_. In this case, both small and large consumers have sufficiently wide ranges of potential prey size. If we assume that the biomass within each imaginary resource size class is equal (*i*.*e*., the resource spectrum is characterized by a constant slope –1), then we can calculate *φ*_*i*,1_ as the sum of *φ*_*i*,*x*_ over all integers *x* in the range [–*s*, 1]. Thus,

$$\varphi_{i,j}=\left\{ \begin{array}{ll} \exp\left[-{\left(\ln\frac{\beta_{i}m_{j}}{m_{i}}\right)}^{2}/2\sigma^{2}\right] & \mathrm{if}\;j>1\\ \sum_{x=-s}^{1}\exp\left[-{\left(\ln\frac{\beta_{i}m_{x}}{m_{i}}\right)}^{2}/2\sigma^{2}\right] & \mathrm{if}\;j=1 \end{array}\right.$$ (3)

where *β*_*i*_ is the most preferred PPMR for a predator individual in size class *i* given by $\upsilon m_{i}^{\tau }$, indicating that if *τ* is equal to 0, then *β*_*i*_ is a constant value; otherwise, *β*_*i*_ scales allometrically with *m*_*i*_, and *σ* is the width (standard deviation) of this function, determining the range of prey sizes that a predator individual prefers. (For the list of parameter definitions, see Table 1.)

**Table 1:** Parameter values used in the basic model simulation case.

Parameter	Definition	value	Source
*γ*	Factor of searching rate	600 m^-3^ yr^-1^ g^-*p*^	[23]
*p*	Exponent of searching rate	0.75	[13]
*υ*	Factor of preferred PPMR	100	[22]
*Τ*	Exponent of preferred PPMR	0	[22]
*σ*	Width of size preference	1.3	[22]
*v*	Factor of standard metabolic rate	2.4 yr^-1^ g^1-*q*^	[13]
*q*	Exponent of standard metabolic rate ^a^	0.75	[24]
*μ*_0_	Factor of non-predation mortality	0.6 yr^-1^ g^-*d*^	[11]
*d*	Exponent of non-predation mortality	0.25	[19]
*k*_*s*_	Factor of carrying capacity ^a^	0.0015	Modified from [4]
*f*	Exponent of carrying capacity ^b^	1.15	Modified from [4]
*c*	Factor of reproduction rate ^c^	10 yr^-1^ g^1-*r*^	Estimates from [6]
*r*	Exponent of reproduction rate ^d^	0.75	
*α*	Sex ratio ^e^	0.5	Arbitrary
*e*	Assimilation efficiency ^f^	0.6	[13]
*q*_0_	Catchability coefficient	0.1	Arbitrary
Δ*t*	Time step ^g^	0.001 yrs	
*δ*	Mass ratio of successive size classes	1.5	

^a^Adjusted from the factor of natural mortality rate in [4] to output a linear size spectrum.

^b^Adjusted from the exponent of natural mortality rate in [4] to output a linear size spectrum.

^c^Estimated from the fraction of the assimilated energy allocated to reproduction.

^d^We use the value *r = p = q =* 0.75 assuming that the reproduction rate is proportional to the searching rate and the standard metabolic rate.

^e^The proportion of females (sex ratio) is assumed to be constant across all size classes, *i*.*e*., *α*_1_ = *α*_2_ = ⋯ = *α*_*k*_.

^f^Individuals in each size class *i* are assumed to have the same assimilation efficiency, *i*.*e*., *e*_1_ = *e*_2_ = ⋯ = *e*_*k*_.

^g^It should be ensured that the probability of each individual growing up to its next size class can be no larger than one in each time step.

Suppose that the volume of water searched by a predator per unit time Δ*t* is expressed as an allometric function of predator body mass *γm*^*p*^Δ*t* [25] and that the encounter rate of each predator individual to prey in size class *j* is also proportional to the number of individuals in prey size class *j*. Then, the total number of individuals in a prey size class *j* eaten by predators (∑_*i*_*P*_*i*,*j*_) per unit time Δ*t* can be expressed as

$$\Delta n^{P-predation}=\left(\begin{array}{c} \sum_{i}P_{i,1}\\ \sum_{i}P_{i,2}\\ \vdots \\ \sum_{i}P_{i,k-1}\\ \sum_{i}P_{i,k} \end{array}\right)=NS^{T}Vn\Delta t=\left(\begin{array}{ccccc} 0 & n_{1}\gamma m_{2}^{p}\varphi_{2,1} & \cdots & n_{1}\gamma m_{k-1}^{p}\varphi_{k-1,1} & n_{1}\gamma m_{k}^{p}\varphi_{k,1}\\ 0 & 0 & \cdots & n_{2}\gamma m_{k-1}^{p}\varphi_{k-1,2} & n_{2}\gamma m_{k}^{p}\varphi_{k,2}\\ \vdots & \vdots & \ddots & \ddots & \vdots \\ 0 & 0 & \cdots & 0 & n_{k-1}\gamma m_{k}^{p}\varphi_{k,k-1}\\ 0 & 0 & \cdots & 0 & 0 \end{array}\right)\left(\begin{array}{c} n_{1}\\ n_{2}\\ \vdots \\ n_{k-1}\\ n_{k} \end{array}\right)\Delta t$$ (4)

where **N** = diag(**n**), **S**^T^ is the transpose of matrix **S**, and $V=\mathrm{diag}(\gamma m_{1}^{p},\gamma m_{2}^{p},\ldots ,\gamma m_{k}^{p})$, describing the search rate of each predator individual in size class *i*. Note that the elements on the diagonal matrix **N** represent the number of prey individuals in each size class, whereas the entries of **n** represent the number of predator individuals.

On the other hand, the total biomass of individuals assimilated by predators in size class *i* with an assimilation efficiency *e*_*i*_ (*e*_*i*_∑_*j*_*m*_*j*_*P*_*i*,*j*_) per unit time Δ*t* is calculated as

$$\left(\begin{array}{c} e_{1}\sum_{j}m_{j}P_{1,j}\\ e_{2}\sum_{j}m_{j}P_{2,j}\\ e_{3}\sum_{j}m_{j}P_{3,j}\\ \vdots \\ e_{k}\sum_{j}m_{j}P_{k,j} \end{array}\right)=ENVSMn\Delta t=\left(\begin{array}{ccccc} 0 & 0 & 0 & \cdots & 0\\ e_{2}n_{2}\gamma m_{2}^{p}\varphi_{2,1}m_{1} & 0 & 0 & \cdots & 0\\ e_{3}n_{3}\gamma m_{3}^{p}\varphi_{3,1}m_{1} & e_{3}n_{3}\gamma m_{3}^{p}\varphi_{3,2}m_{2} & 0 & \cdots & 0\\ \vdots & \vdots & \ddots & \ddots & 0\\ e_{k}n_{k}\gamma m_{k}^{p}\varphi_{k,1}m_{1} & e_{k}n_{k}\gamma m_{k}^{p}\varphi_{k,2}m_{2} & e_{k}n_{k}\gamma m_{k}^{p}\varphi_{k,3}m_{3} & \cdots & 0 \end{array}\right)\left(\begin{array}{c} n_{1}\\ n_{2}\\ n_{3}\\ \vdots \\ n_{k} \end{array}\right)\Delta t$$ (5)

where **E** = diag(*e*_1_,*e*_2_,…,*e*_*k*_), whose entry *e*_*i*_ describes the assimilation efficiency of individuals in each predator size class *i* and **M** = diag(**m**) represents the prey body mass. Note that, in this case, the diagonal matrix **N** represents the number of predator individuals, while the vector **n** represents the number of prey individuals, which is different from the case of Eq (4). The first entry of the product **ENVSMn** becomes zero, which means that resources conceptually do not prey on others.

Increases in the body mass of predators in size class *i* owing to assimilation of prey can cause a certain number of predator individuals *G*_*i*_ to grow to the next size class *i*+1. According to the discretization of the McKendrick-von Foerster equation (see S1 File), we assume that *G*_*i*_ = *e*_*i*_∑_*j*_*m*_*j*_*p*_*i*,*j*_/(*m*_*i*+1_ − *m*_*i*_), which means that the number of individuals transferred to size class *i*+1 is calculated by dividing the total biomass assimilated in size class *i* by the difference in individual masses between the neighboring size classes *i* and *i*+1. Then,

$$\left(\begin{array}{c} G_{1}\\ G_{2}\\ G_{3}\\ \vdots \\ G_{k} \end{array}\right)=\Delta M_{+}^{-1}ENVSMn\Delta t=\left(\begin{array}{ccccc} 0 & 0 & 0 & \cdots & 0\\ \frac{e_{2}n_{2}\gamma m_{2}^{p}\varphi_{2,1}m_{1}}{m_{3}-m_{2}} & 0 & 0 & \cdots & 0\\ \frac{e_{3}n_{3}\gamma m_{3}^{p}\varphi_{3,1}m_{1}}{m_{4}-m_{3}} & \frac{e_{3}n_{3}\gamma m_{3}^{p}\varphi_{3,2}m_{2}}{m_{4}-m_{3}} & 0 & \cdots & 0\\ \vdots & \vdots & \ddots & \ddots & 0\\ \frac{e_{k}n_{k}\gamma m_{k}^{p}\varphi_{k,1}m_{1}}{m_{k+1}-m_{k}} & \frac{e_{k}n_{k}\gamma m_{k}^{p}\varphi_{k,2}m_{2}}{m_{k+1}-m_{k}} & \frac{e_{k}n_{k}\gamma m_{k}^{p}\varphi_{k,3}m_{3}}{m_{k+1}-m_{k}} & \cdots & 0 \end{array}\right)\left(\begin{array}{c} n_{1}\\ n_{2}\\ n_{3}\\ \vdots \\ n_{k} \end{array}\right)\Delta t$$ (6)

where matrix Δ**M**_+_ = diag(*m*_2_ − *m*_1_,*m*_3_ – *m*_2_,…,*m*_*k*+1_ − *m*_*k*_) describes the mass difference between the upper (*i*.*e*., subscript “+”) size class *i*+1 and size class *i*, and $\Delta M_{+}^{-1}$ is the inverse of matrix Δ**M**_+_.

According to the growth process per unit time Δ*t* in Fig 1A, *G*_*i*−1_ individuals from size class *i*–1 enter size class *i*, while *G*_*i*_ individuals in size class *i* leave size class *i*, resulting in a net change in the number of individuals in each size class *i* given of *G*_*i*−1_ – *G*_*i*_. Additionally, there is an increase in the number of individuals in size class 1 corresponding to the recycling of carrion *C*_*k*+1_ from imaginary size class *k*+1, which is given by *C*_*k*+1_/*m*_1_ (or *G*_*k*_*m*_*k*+1_/*m*_1_). As the dimension of *C*_*k*+1_ is mass per unit time, division by *m*_1_ converts the mass into the number of resources. Thus, shifts in the number of individuals (Δ**n**^*G*^) caused by growth and the recycling of carrion from size class *k*+1 per unit time Δ*t* are expressed as follows:

$$\Delta n^{G}=(T_{g}+T_{c})\Delta M_{+}^{-1}ENVSMn\Delta t$$ (7)

where

$$T_{g}=\left(\begin{array}{ccccc} -1 & 0 & 0 & \cdots & 0\\ 1 & -1 & 0 & \cdots & 0\\ 0 & 1 & -1 & \cdots & 0\\ \vdots & \vdots & \ddots & \ddots & \vdots \\ 0 & 0 & 0 & \cdots & -1 \end{array}\right)$$ (8)

is a transfer matrix, with entries –1 on the diagonal and 1 on the subdiagonal, for transferring *G*_*i*_ to the next size class, and

$$T_{c}=\left(\begin{array}{ccccc} 0 & 0 & 0 & \cdots & \frac{m_{k+1}}{m_{1}}\\ 0 & 0 & 0 & \cdots & 0\\ 0 & 0 & 0 & \cdots & 0\\ \vdots & \vdots & \ddots & \ddots & \vdots \\ 0 & 0 & 0 & \cdots & 0 \end{array}\right)$$ (9)

is a matrix to transfer carrion from size class *k+*1 to be recycled in size class 1.

In addition to the assimilated biomass in Eq (5), the total undigested (unassimilated) materials *E*_*i*_ per unit time Δ*t* of each size class *i* is given by (1−*e*_*i*_)∑_*j*_*m*_*j*_*P*_*i*,*j*_. This can be equivalently written in matrix form as (**I−E)NVSMn**, where **I** is a unit matrix with ones on the diagonal and zeros elsewhere. For the recycling of these undigested materials into resources, a new transition matrix **T**_1_ to size class 1 is introduced:

$$\Delta n^{E}=T_{1}M_{1}^{-1}(I-E)NVSMn\Delta t$$ (10)

where Δ**n**^*E*^ denotes the increase in the number of resources caused by recycling of undigested (unassimilated) materials per unit time Δ*t*,

$$T_{1}=\left(\begin{array}{ccccc} 1 & 1 & 1 & \cdots & 1\\ 0 & 0 & 0 & \cdots & 0\\ 0 & 0 & 0 & \cdots & 0\\ \vdots & \vdots & \vdots & \ddots & \vdots \\ 0 & 0 & 0 & 0 & 0 \end{array}\right),$$ (11)

and $M_{1}^{-1}$ is the inverse of matrix **M**_1_ = diag(*m*_1_,*m*_1_,…,*m*_1_). Multiplying (**I−E)NVSMn** by $M_{1}^{-1}$ converts *E*_*i*_ in Fig 1A, whose dimension is mass per unit time, into number of resources; multiplication by **T**_1_ then transfers all undigested materials from each size class *i* to size class 1.

In conclusion, by combining Eqs (4), (7) and (10), we obtain Δ**n**^*P*^ as the shift in numbers obtained from the predator–prey interaction process per unit time Δ*t*:

$$\begin{array}{l} \Delta n^{P}=-\Delta n^{P-predation}+\Delta n^{G}+\Delta n^{E}\\ \;=(-NS^{T}V+((T_{g}+T_{c})\Delta M_{+}^{-1}E+T_{1}M_{1}^{-1}(I-E))NVSM)n\Delta t \end{array}$$ (12)

#### Reproduction

As depicted in Fig 1B, reproduction occurs only in the size range [*m*_3_,*m*_*k*_]. We assume that the mean proportions of females and males in each size class *i* are *α*_*i*_ and 1−*α*_*i*_, respectively. The reproduction rate *F*_*i*_ is given by $cm_{i}{}^{r}\Delta t$, where *c* is the total biomass of eggs produced by unit weight and *r* is the scaling exponent, which does not differ between sexes.

Newborn individuals (eggs or larvae) are placed in size class 2 and excretive matter (semen) is recycled into size class 1 as newly input resources, thereby causing an increase in the numbers of individuals in size classes 1 and 2 as follows

$$\Delta n^{F}=(T_{1}M_{1}^{-1}(I-A)+T_{2}M_{2}^{-1}A)Rn\Delta t$$ (13)

where **A** = diag(*α*_1_,*α*_2_,…,*α*_*k*_) is a sex ratio matrix, $R=\mathrm{diag}(0,\;0,cm_{3}^{r},\ldots ,cm_{k}^{r})$ is the reproduction rate matrix, **T**_2_ is a new matrix for transferring eggs into size class 2, where

$$T_{2}=\left(\begin{array}{ccccc} 0 & 0 & 0 & \cdots & 0\\ 1 & 1 & 1 & \cdots & 1\\ 0 & 0 & 0 & \cdots & 0\\ \vdots & \vdots & \vdots & \ddots & \vdots \\ 0 & 0 & 0 & \cdots & 0 \end{array}\right),$$ (14)

and $M_{2}^{-1}$ is the inverse of matrix **M**_2_ = diag(*m*_2_,*m*_2_,…,*m*_2_).

Assuming that *B*_*i*_ individuals per unit time Δ*t* are transferred from size class *i* to the next-lower size class *i*–1 owing to mass decrease by reproduction and is calculated using $cm_{i}^{r}n_{i}/\left(m_{i}-m_{i-1}\right)$. The loss in the number of individuals in size class *i* as a result of transfer to size class *i*–1 and the gain in the number of individuals in size class *i* as a result of transfer from size class *i*+1 lead to a net change −*B*_*i*_+*B*_*i*+1_ in the number of individuals in each size class *i* be given by

$$\Delta n^{B}=T_{g}^{T}\Delta M_{-}^{-1}Rn\Delta t$$ (15)

where Δ**n**^*B*^ denotes the shift in the number of individuals as a result of this process and $\Delta M_{-}^{-1}$ is the inverse of matrix Δ**M**_ = diag(*m*_1_−*m*_0_,*m*_2_−*m*_1_,…,*m*_*k*_−*m*_*k*−1_) (where *m*_0_ = 0) that describes the mass difference between size class *i* and the lower (hence, the subscript “−”) size class *i*–1.

Therefore, the column vector Δ**n**^*R*^ is given by adding Eqs (13) and (15):

$$\begin{array}{l} \Delta n^{R}=\Delta n^{F}+\Delta n^{B}\\ \;=(T_{1}M_{1}^{-1}(I-A)+T_{2}M_{2}^{-1}A+T_{g}^{T}\Delta M_{-}^{-1})Rn\Delta t \end{array}$$ (16)

#### Metabolism

Individuals must pay metabolic costs to maintain body structure and activity. Standard metabolic rate is recognized as an allometric function of body mass: *vm*^*q*^Δ*t* [24]. As shown in Fig 1C, the model of metabolism is similar to that of reproduction. Although resources do metabolize, the metabolites are eventually recycled back to their own size class, resulting in no variation in the number of individuals. Therefore, for simplicity only the metabolic costs of the consumers’ group are considered in our model. Recycling of these metabolites leads to an increase in the number of individuals in size class 1 as follows:

$$\Delta n^{W}=T_{1}M_{1}^{-1}Qn\Delta t$$ (17)

where $Q=\mathrm{diag}(0,vm_{2}^{q},\ldots ,vm_{k}^{q})$ is the metabolic rate matrix whose diagonal elements $vm_{i}^{q}$ are the metabolic rates *W*_*i*_ of the individuals in size class *i*.

If we assume that number of individuals transferred from size class *i* to the next-lower size class *i*–1 is given by $L_{i}=vm_{i}^{q}n_{i}/\left(m_{i}-m_{i-1}\right)$, then the net change −*L*_*i*_+*L*_*i*+1_ in the number of individuals in each size class *i* per unit time Δ*t* is given by

$$\Delta n^{L}=T_{g}^{T}\Delta M_{-}^{-1}Qn\Delta t$$ (18)

where Δ**n**^*L*^ denotes the shift in the number of individuals as a result of this process.

Therefore, the resulting column vector Δ**n**^*Q*^ is the sum of Eqs (17) and (18):

$$\begin{array}{l} \Delta n^{Q}=\Delta n^{W}+\Delta n^{L}\\ \;=(T_{1}M_{1}^{-1}+T_{g}^{T}\Delta M_{-}^{-1})Qn\Delta t \end{array}$$ (19)

#### Non-predation and fishing mortalities

The causes of non-predation mortality include disease, competition, senescence, and other natural factors. McCoy and Gillooly [19] stated that instantaneous natural mortality rate scales allometrically with body mass as *μ*_0_*m*^−*d*^. However, the predation mortality for the largest individuals in our model is zero, while non-predation mortality is also naturally very low, resulting in an excessive accumulation of individuals. Increasing or decreasing the mortality of top-predators can cause a top-down cascade that leads to a change in the size distribution of their prey individuals and produces a wave-like biomass spectrum [26].

To obtain a linear size spectrum, we modulate the mortality of large individuals by introducing a density-dependent mortality as in Benoît and Rochet [4] but with the density-dependent mortality increasing with the ratio between the number of individuals and the carrying capacity. We assume that the carrying capacity is size-based and given by *k*_*s*_*m*^−*f*^, where *k*_*s*_ is the carrying capacity factor indicating the maximum number of individuals that the community can support per unit mass and *f* is the scaling exponent. Consequently, the non-predation mortality of each individual in size class *i* becomes $n_{i}z_{0}m_{i}^{-z}$, where *z*_0_ = *μ*_0_/*k*_*s*_ and *z* = *d*−*f*.

Thus, the decreased number of individuals *D*_*i*_ owing to non-predation mortality (Δ**n**^*D−loss*^) in each size class *i* per unit time Δ*t* is

$$\Delta n^{D-loss}=NDn\Delta t$$ (20)

where the product $ND=\mathrm{diag}(0,n_{2}z_{0}m_{2}^{-z},\ldots ,n_{k}z_{0}m_{k}^{-z})$, whose diagonal element $n_{i}z_{0}m_{i}^{-z}$ gives the non-predation mortality rate of each individual in size class *i*.

The recycling of carrion from each size class *i* yields new production (Δ**n**^*D−recycle*^) in size class 1:

$$\Delta n^{D-recycle}=T_{1}M_{1}^{-1}NDMn\Delta t$$ (21)

where the entries of the product **NDMn** give the biomass of the carrion of each size class *i*.

Fishing can affect community structures in marine ecosystems. Several community size spectrum models and trait-based size spectrum models have been used to investigate the effect of fishing [4, 11–13], and fishing mortality can be easily included in this matrix model to examine the effects of different fishing patterns on the size spectrum. Similar to the process of non-predation mortality, the decrease in the number of individuals as a result of fishing is given as

$$\Delta n^{Y-remove}=Hn\Delta t$$ (22)

where **H** = diag(*h*_1_,*h*_2_,…,*h*_*k*_) is the fishing mortality rate matrix, whose diagonal element gives the fishing mortality rate of each individual in size class *i*. The recycling of these catches (Δ**n**^*Y−recycle*^) through human consumption into size class 1, as depicted in Fig 1D, is then expressed as

$$\Delta n^{Y-recycle}=T_{1}M_{1}^{-1}HMn\Delta t$$ (23)

Finally, combining Eqs (20), (21), (22) and (23), we obtain:

$$\begin{array}{l} \Delta n^{U}=-\Delta n^{D-loss}+\Delta n^{D-recycle}-\Delta n^{Y-remove}+\Delta n^{Y-recycle}\\ \;=(-ND-H+T_{1}M_{1}^{-1}(ND+H)M)n\Delta t \end{array}$$ (24)

#### Unified size-structured matrix model

The overall matrix model for expressing size-structured community dynamics can be obtained by adding Eqs (12), (16), (19) and (24):

$$\begin{array}{l} n(t+\Delta t)=n(t)+\Delta n^{P}+\Delta n^{R}+\Delta n^{Q}+\Delta n^{U}\\ \;=n(t)-(NS^{T}V-(T_{g}+T_{c})\Delta M_{+}^{-1}ENVSM+ND+H\\ \;-T_{1}M_{1}^{-1}((I-E)NVSM+(I-A)R+Q+(ND+H)M)\\ \;-T_{2}M_{2}^{-1}AR-T_{g}^{T}\Delta M_{-}^{-1}(R+Q))n(t)\Delta t \end{array}$$ (25)

### Parameter settings for numerical simulation

The egg size of marine fish is fairly constant among species and lies predominantly within a narrow range around 1 mg [27]. Thus, we set the mass of the first consumer size class as *m*_2_ = 0.001 g. To obtain a smooth line, we use a small value for the mass ratio of successive size classes, *δ*. The size range of imaginary resources [*m*_−*s*_,*m*_0_] is also discretized using the ratio *δ*: *m*_*x*+1_ = *m*_*x*_*δ*, where *x* = −*s*, −*s* + 1…,0. This range roughly extends from *m*_−*s*_ = 1.36×10^−10^ g to *m*_0_ = 4.44×10^−4^ g, which also covers very small phytoplankton cells [28]. To cover the size range of large adult fish in the ocean, the size axis is discretized over 50 size classes ranging from *m*_1_ = 6.67×10^−4^ g to *m*_*k*_ = 2.83×10^5^ g. We assume that the total biomass of the community is 0.5 g/m^-3^ and run the model starting from an initial condition $n_{i}=0.01m_{i}^{-1}$ until it reaches a stationary state for each numerical simulation. The simulations are performed in Matlab (see code in S2 File). The steady-state conditions for size spectra are achieved within a 40-year model run (*i*.*e*., Eq (25)). It has been determined that the results do not depend on the initial number of individuals within each size class.

The other parameter values used in the basic model simulation case are listed in Table 1. Most of the parameter values are based on previously published size-based models. Parameters such as the exponents of the searching and reproduction rates are given as 3/4 based on the fact that metabolic rate scales to the 3/4 power of body size. The value of the reproduction rate factor *c* is set such that the ratio between the reproduction and assimilation rates falls within the reference range [6]. The values of factor *k*_*s*_ and the exponent *f* of the carrying capacity are derived from modified values of *z*_0_ and *z* in Benoît and Rochet [4].

Increasing the mass ratio *δ* of successive size classes leads to a decrease in the number of size classes; if the number is reduced by half, then it is necessary to increase the number of individuals in each size class to twice the number of individuals in the basic case to keep the total biomass in the community constant. In this case, the carrying capacity factor *k*_*s*_ should also be doubled to support the abundance of individuals. As shown in S1 Fig, varying the value of *δ* does not significantly change the size spectrum. Only the egg size class (*i*.*e*., size class 2) and its adjacent size classes depart slightly from the linear size spectrum because the total energy allocated to reproduction from consumers decreases with increasing value of *δ*. The size spectra obtained with different values of time step Δ*t* are exactly the same (S1 Fig), implying that the time step does not change the dynamics of size spectra. A mass ratio *δ* of successive size classes larger than 1.5 will allow the model to run at a daily or a larger time step (S1 Fig).

### Size-dependency of rates in each process

Using the parameter values in Table 1 results in a mass-specific growth and reproduction rates that decrease with body mass with slopes close to –1/4 (S2 Fig) on a log–log scale. The log–log plot of predation mortality rate versus body size is nearly linear, with a slope close to –1/4 within the size range between 10^−3^ and 10^3^ g. For individuals larger than 10^3^ g, predation mortality decreases sharply with body size owing to the decline in predator individuals (S2 Fig). The logs of non-predation mortality rate and body size show a nearly linear relationship, with higher and lower mortality rates for smaller and larger individuals, respectively, although with a slope that is slightly shallower than –1/4 (S2 Fig). This may be caused by carrying capacity effects.

## Results

### Steady state

**Fig 2:**
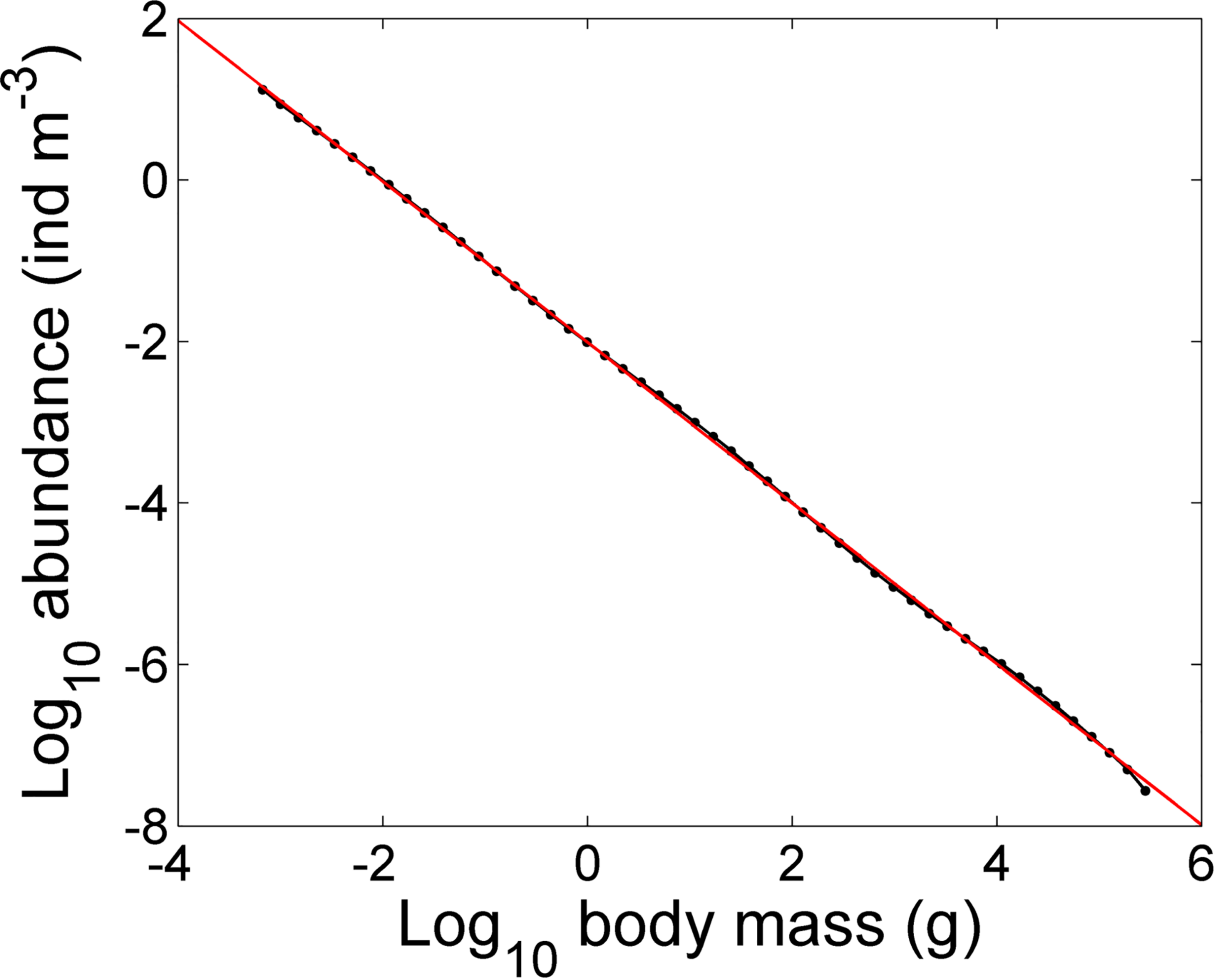
Linear size spectrum. Simulated size spectrum at steady state (dotted line) using reference parameters in Table 1 and a regression line as a function of log body size (solid line; log_10_[abundance] = −0.9959×log_10_[body mass] − 2.006).

Using the reference values of the parameters in Table 1 results in a linear size spectrum with a slope of approximately –1, which is consistent with values reported in empirical studies [4, 6] (Fig 2).

### Examples of numerical simulation

**Fig 3:**
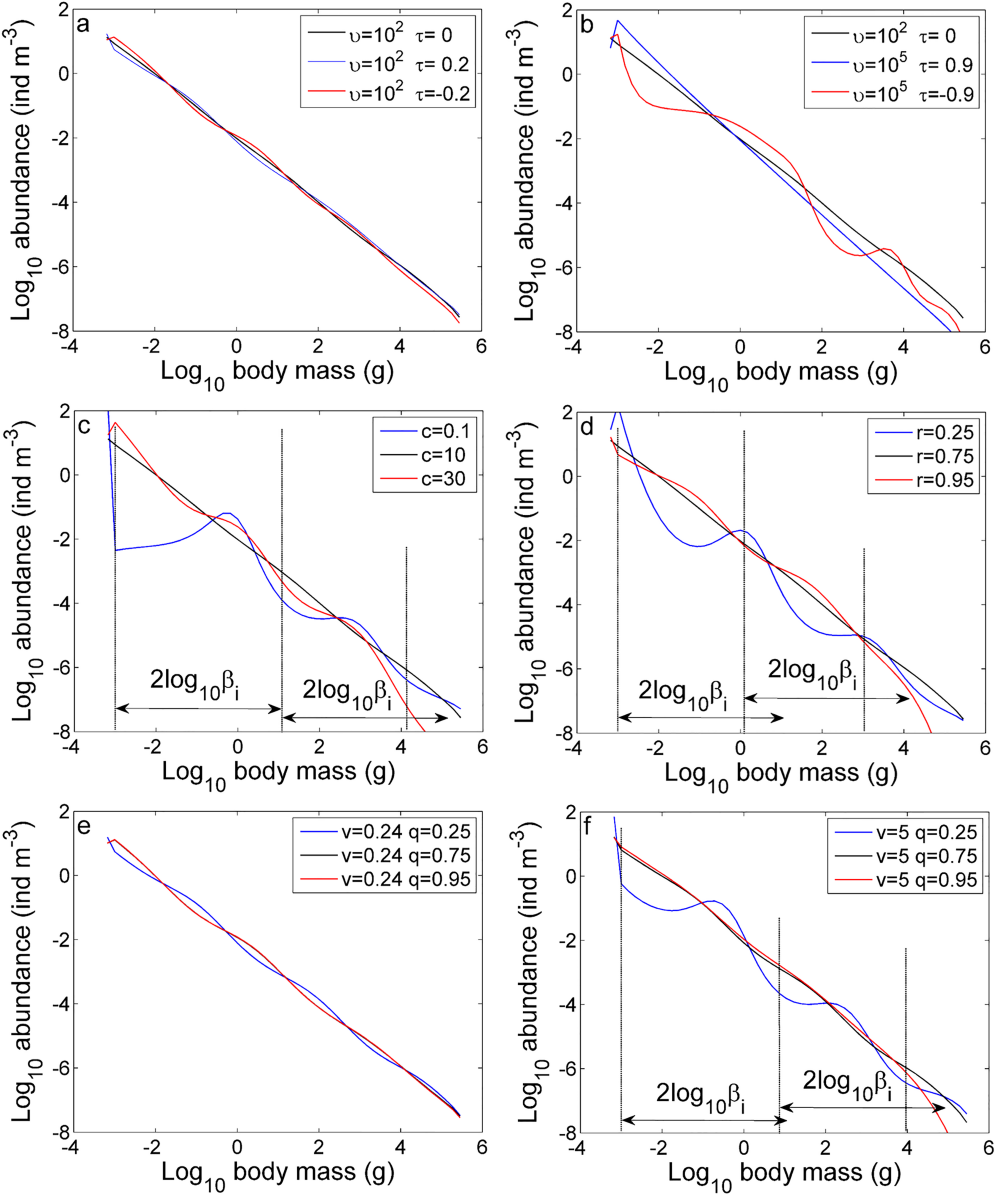
Simulated size spectra at equilibrium with different parameter values. Simulated size spectra using (a) plausible combinations of the factor *υ* and exponent *τ* of PPMR, (b) extreme combinations of *υ* and *τ*, different values of (c) the reproduction rate factor *c*, (d) reproduction rate exponent *r*, (e) metabolic rate exponent *q* with a small metabolic rate factor *v*, and (f) exponent *q* with a large factor *v*. The vertical dotted lines mark wave troughs or peaks and the horizontal distance between two adjacent vertical dotted lines is the wavelength. The horizontal arrows indicate the length of 2log_10_*β*_*i*_.

In the application shown in Fig 2, we simulated the size spectrum with the constant preferred PPMR *β*_*i*_ given in Table 1. However, field data show that there is a great variability in *β*_*i*_ with predator mass [29, 30]. Therefore, the effect of varied *β*_*i*_ ($=\upsilon m_{i}^{\tau }$) was investigated for *τ* ≠ 0. For a plausible range of *τ* (−0.2 ≤ *τ* ≤ 0.2), the size spectra are essentially identical to the basic case spectrum (Fig 3A). An extreme range of *τ* (−0.9≤*τ*≤0.9), which apparently exceeds the plausible range obtainable in an actual situation, was also examined to obtain a clear contrast in terms of the effect of *τ*. For *υ* = 10^5^ and *τ* = −0.9, for which *β*_*i*_ ranges from approximately 5×10^7^ for small predators of mass 10^−3^ g to approximately 1.0 for the largest predators of mass 3×10^5^ g, static waves appear along the size spectrum (Fig 3B). By contrast, for *υ* = 10^5^ and *τ* = 0.9, for which *β*_*i*_ increases with predator body mass and ranges from approximately 2×10^2^ for small predators of 10^−3^ g to approximately 8×10^9^ for the largest predators, the resulting size spectrum shows a slightly steeper slope (Fig 3B). Under various combinations of *υ* and *τ*, the wavelengths of the size spectra vary (S3 Fig). For all cases the slopes range from approximately –0.98 to –1.16 but are close to that of the basic case shown in Fig 2 (ca. –1). Thus, except for the wavelengths along the size spectrum, PPMR does not significantly affect the size spectrum.

Reduction in the value of *c* or *r* in the reproductive rate $cm_{i}^{r}$ leads to a decrease in reproductive allocation and an eventual accumulation of large individuals, which in turn can cause the population of their most preferred prey to decrease through predation as a top-down effect, resulting in the alternate propagation of opposed effects to smaller size ranges. As a result, waves propagate through the size spectrum (S4 Fig). Size spectra with static waves emerge when the system reaches equilibrium conditions (Fig 3C and 3D). The wavelength depends on PPMR [31], with observed wavelengths approximately equal to or less than 2log_10_*β*_*i*_ on a log–log scale (Fig 3C and 3D). Conversely, increasing the value of *c* or *r* leads to a decrease in the number of large individuals and a cut-off of the size spectrum (Fig 3C and 3D), which arises because large individuals convert more energy into reproduction, leaving less energy available for growth.

The standard metabolic rate *vm*^*q*^ is smaller than both the predation and reproduction rates and therefore might have a weak effect on the size spectrum. To show how the size spectrum can be affected by the standard metabolic rate, we set the factor *v* at different levels for comparison. For low values of *v* (Fig 3E), the standard metabolic rate of each individual in size class *i* is very small, and therefore changing the exponent *q* has no apparent effect on the size spectrum. For large values of *v*, however, varying *q* changes the shape of the size spectrum (Fig 3F). A size spectrum with static waves with wavelengths less than 2log_10_*β*_*i*_ appears when *q* decreases from the reference value 0.75 to a value of 0.25. Before reaching an equilibrium state, waves travel from small to large individuals (S4 Fig). An increase in *q* produces a size spectrum with a cut-off in the range of large body masses.

### Effect of fishing on the size spectrum

Two types of fishing patterns were compared: (i) an unbalanced fishing pattern in which individuals larger than 10^4^ g are caught with a constant fishing mortality *q*_0_*F*_0_, where *q*_0_ is the catchability coefficient and *F*_0_ is the fishing intensity coefficient; and (ii) a balanced fishing pattern in which individuals larger than 10 g are caught in proportion to their natural productivity determined by the metabolic size-scaling rules [18, 32], *i*.*e*., the fishing mortality rate for individuals in this size range is given by $q_{0}F_{0}m_{i}^{-0.25}$. The fishing mortality rate matrices corresponding to fishing patterns (i) and (ii) are given by **H**_1_ = diag(0,…,0, *q*_0_*F*_0_,…,*q*_0_*F*_0_) and $H_{2}=\mathrm{diag}(0,\ldots ,0,q_{0}F_{0}m_{i}^{-0.25},\ldots ,q_{0}F_{0}m_{k}^{-0.25})$, respectively (S5 Fig).

**Fig 4:**
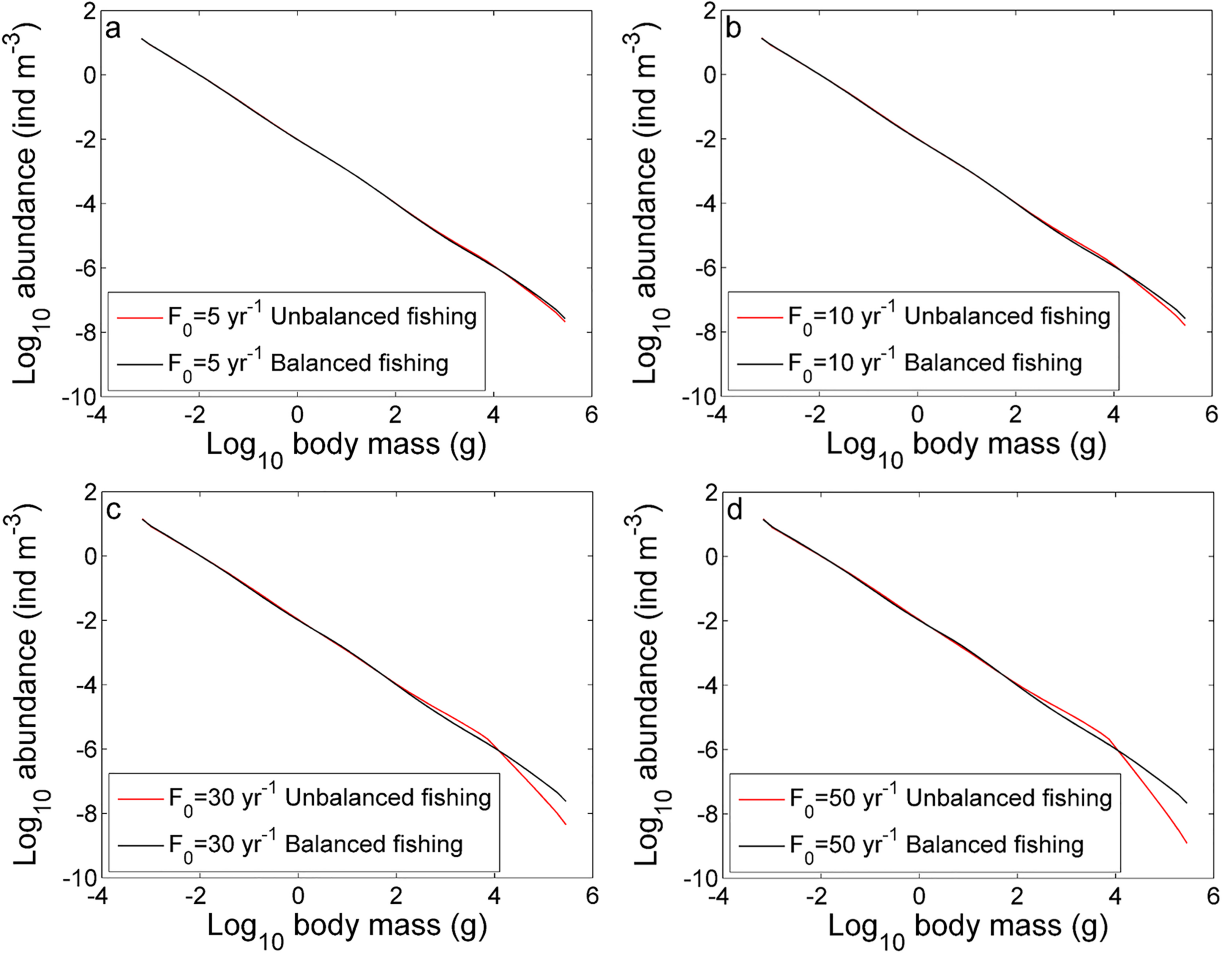
Predicted impacts of fishing. Effects of unbalanced and balanced fishing on the size spectrum at different fishing intensity coefficient levels: (a) *F*_0_ = 5 yr^−1^, (b) *F*_0_ = 10 yr^−1^, (c) *F*_0_ = 30 yr^−1^, and (d) *F*_0_ = 50 yr^−1^.

The unbalanced fishing pattern results in a more severe cut-off when the fishing intensity coefficient *F*_0_ increases from 5 yr^-1^ to 50 yr^-1^; by contrast, the balanced fishing pattern does not result in a cut-off of the spectrum even when the fishing intensity coefficient increases (Fig 4). Yields from the balanced fishing pattern are much higher than those of the unbalanced fishing pattern (S6 Fig).

## Discussion

We developed a size-structured matrix model to express community dynamics in which each process is modeled by simple body size power functions. The model can be seen as a discretization of the Mckendrick-von Foerster partial differential equation that produces a diffusion of growth, *i*.*e*., during each time step, several individuals grow up to the next-higher size class while other individuals remain in the same size class. Thus, the basic difference between our matrix model and the continuous Mckendrick-von Foerster equation lies in its ability to incorporate growth variation in individuals within a size class; in a real process, this diffusion is appropriate because individuals with the same size in a given population often differ in growth rate [33]. The main advantage of our model lies in its flexibility in modeling each process. In addition, the model can be run on a daily or larger time step Δ*t* by increasing the mass ratio *δ* of successive size classes. In this case, the running time for numerical simulations required by our matrix model will be much shorter than that of PDE models as a result of the reduced number of size classes and loop iterations.

Boundary conditions imposed in upper and lower size classes can affect the shapes of size spectra. For the upper boundary, the introduction of senescence mortality in previous models [14, 34–36] led to a sharp increase in the mortality of large individuals. As a result, large size classes depart from the linear size spectrum, resulting in a downwardly curved stationary size spectrum and the emergence of a cut-off. However, field data support the assertion that natural mortality for organisms is negatively correlated with body mass [19, 37]. We therefore adopt an allometric natural mortality rate against body mass [11] based on empirical information. Our model is developed with a type I functional response, which can produce an unlimited growth of predators and an unrealistic increase in the number of these individuals. Introducing a density-dependent effect to the allometric natural mortality rate can help control the increase in predator individuals, thereby improving the stability of size spectra.

For the lower boundary, the growth and reproduction of background resources based on the processes of nutrient uptake and photosynthesis are not explicitly modeled in our study; nevertheless, our model can successfully reproduce linear size spectra with slopes close to –1 (Fig 2). The size spectrum produced by the basic case does not depart from a linear shape for nearly all size classes, possibly because we introduced an imaginary size range to allow consumers to have a common food availability level.

Under the mass balance assumption, variation in the biomass (and abundance) of background resources is controlled by the difference between the rates of recycling of biomass egested from consumers and consumption of resource biomass by predators. In our model, the community can reach equilibrium conditions even under extreme parameter values, although size spectra with cut-offs and/or static waves are frequently observed in such cases (Fig 3). If the biomass of resources is assumed to be constant [14, 31], infinite oscillations occur when the non-predation mortality rate is set to zero, while only transient oscillations emerge in this case under the mass balance assumption (S7 Fig). Under the constant resource assumption, there are some cases in which the total biomass of the community will continue to decrease, eventually leading to a collapse of the community under some extreme parameter value assumptions, *e*.*g*., *r* = 0.25 (S8 Fig). Owing to the mass balance assumption, the effect of extreme parameter values on the size spectrum can be alleviated. If model parameters are varied over reasonable ranges, similar results can still be obtained (S8 Fig). However, because carbon is exchanged and recycled throughout the biosphere and the mass balance in a local community might not always be retained, it will be important to examine the consequences of various types of assumptions on background resources in future studies that explore the dynamics of real marine communities.

Maury *et al*. [6] found that their model produces unstable oscillations when the reproductive investment of females is extremely low. Increasing the value *β*_*i*_ of the most preferred PPMR or decreasing the feeding kernel width *σ* can also make the size spectrum unstable and generate traveling waves (*e*.*g*., [31]). Unlike these previous studies, neither infinite oscillations nor traveling waves appeared in our study. In addition to the mass balance assumption discussed above, this may be because of the presence of growth diffusion in our model, which can help stabilize the size spectrum [14].

Thiebaux and Dickie [38] mathematically modeled a predator biomass spectrum with a parabolic dome and predicted that the related prey biomass spectrum would also become a parabola as a result of energy transfer between trophic levels, which explained their corresponding wave-like size spectra. Our results suggest that such waves are caused by the accumulation of large individuals, as a top-down cascade can be stimulated by an increase in the number of predators with mass *m*_*k*_ in the largest size class suppressing their prey individuals with mass *m*_*k*_/*β*_*k*_, which in turn releases individuals with mass $m_{k}/\beta_{k}^{2}$ from predation. This should result in a wavelength approximately equal to 2log_10_*β*_*i*_ on a log–log scale; however, our results primarily produced wavelengths less than 2log_10_*β*_*i*_. This may have been caused by growth rates that differed between predators and their preferred prey: in this case, because small individuals travel faster than large individuals along the size spectrum, the distance between the peak of one wave and the next-lower trough would become smaller than PPMR.

Wave-like size spectra have also been observed in nature. For example, Gaedke [39] reported wave-like oscillations in the seasonal distribution of the biomass of individuals ranging from bacteria to crustaceans. These results imply that a simple linear size spectrum will not be sufficient to describe the dynamics of actual marine communities. Wave-like oscillations also need to be recognized as important indicators of ecosystems, and if simple linear regression analysis alone is relied upon, the presence of cut-offs and/or waves might result in inadequate slope estimates. The shape of a size spectrum should therefore be looked at closely in revealing the impacts of human activities and environmental changes.

To show the applicability of our model, a short example concerning the fishing effects on a marine community was enumerated (see the last part of Results). The present matrix model can be easily expanded to enable investigation of the effects of various other situations, *e*.*g*., stochastic or periodic environmental changes such as water temperature and productivity, which vary with season [40]. Other processes such as seasonal reproduction and long-term environmental changes can also be coupled to the model to investigate the response of marine community size spectra at large scales.

The model can be easily modified to take species identity into consideration and include population demography. To achieve this, we can replace the column vector **n**(*t*) with a new column vector, *e*.*g*., **n**_(*sp*.1)_(*t*) to represent the structure of species 1. For instance, in the predator–prey interaction process the total number of individuals of species 1 in each size class eaten by predators of other species *h* (h=1,2,…,l) per unit time Δ*t* can be expressed as $\sum_{h=1}^{h=l}N_{\left(sp.h\right)}S^{T}\theta_{\left(sp.h,1\right)}Vn_{\left(sp.1\right)}$, where **N**_(*sp*.*h*)_ is the abundance matrix of species *h* and **θ**_(*sp*.*h*,1)_ is a matrix representing the interaction strength between species *h* and 1. The elements in the interaction strength matrix can be freely varied for application to several cases, *e*.*g*., in which species’ prey preferences vary by prey species and size. Likewise, species-specific reproduction can be modeled by setting the maturation and maximum sizes in each species’ abundance vector (*e*.*g*., **n**_(*sp*.1)_(*t*) = (*n*_(*sp*.1)1_(*t*), *n*_(*sp*.1)2_(*t*),…, *n*_(*sp*.1)*mature*_(*t*),…, *n*_(*sp*.1)max*imum*_(*t*))^T^). The reproduction rate for each species can then be given by **AR**_(*sp*.*h*)_**n**_(*sp*.*h*)_, where **R**_(*sp*.*h*)_ is a reproduction rate matrix of species *h* whose elements are modified to place the maturation, maximum, and offspring sizes within the proper size classes for the species. Other investigations on, for instance, the impacts of various fishing patterns for different species on marine communities can also be conducted through similar structural modifications of the present model. Consequently, a three-dimensional matrix model formed by three coordinate axes, “body mass” (*x*), “species” (*y*), and “abundance” (*z*), is envisioned.

An important goal for future work is to apply the model to real ecosystem. To produce reasonable results, the model and parameter values in the present study can be calibrated for different ecosystem types based on recorded data related to the vital rates, life histories, and behaviors of individuals within a specific community. Determining appropriate lower and upper boundary conditions will be especially important in calibrating the model to reproduce real-world dynamics. Real size distribution of communities obtained from research vessel data and other sources should be compared with the simulation results derived from the calibrated model through the use of sets of sufficiently powerful statistical measures.

## Conclusion

In this paper, we demonstrated the construction of a community matrix model with several body size power functions from individual-level processes. The model can produce stable size spectra under various conditions. Growth diffusion, which plays a role in stabilizing size spectra, is allowed in our model. When parameter values change, the model produces static waves at equilibrium states as a result of top-down cascades or cut-offs at large size ranges caused by reduction in energy allocated to growth or high fishing mortality.

The matrix algorithms developed in this study can reduce the computational time needed to calculate size spectra. Increasing the mass ratio of successive size classes enables a daily or larger time step without affecting the results. As a result of its flexibility, the model can be easily extended or modified to explore community- or population-level dynamics.

## Supporting information

S1 Fig Influence of mass ratio and time step on the size spectrum at steady state. Normalized size spectra, in which the abundance is divided by the logarithmic width of each size class, at steady states for different values of (a) mass ratio *δ* of successive size classes and (b) time step Δ*t*.
(TIF)

S2 Fig Size-dependent relationships used to parameterize the model. (a) Decrease in mass-specific growth, reproduction, and metabolic rates with increasing size. (b) Decrease in predation mortality, and non-predation mortality rates with increasing size.
(TIF)

S3 Fig Effects of varying PPMR on the size spectrum. Simulated size spectra at steady state for different combinations of factor *υ* and exponent *τ* of PPMR.
(TIF)

S4 Fig Examples of transient oscillations prior to reaching equilibrium. When (a) the reproduction rate factor *c* is set to 0.1 and (b) the metabolic rate factor *v* and exponent *q* are set to 5 and 0.25, respectively, the model produces transient oscillations until the system reaches a stationary state. Dotted lines represent the waves propagating through the spectrum, while solid lines represent the stationary states (*i*.*e*., static waves).
(TIF)

S5 Fig Fishing mortality. Relationship between body mass and fishing mortality for four different fishing intensity coefficients *F*_0_ under unbalanced and balanced fishing patterns.
(TIF)

S6 Fig Yields from the two fishing patterns under different fishing intensity coefficients. (TIF)

S7 Fig Comparison of equilibrium and non-equilibrium states. When the non-predation mortality rate factor *μ*_0_ is set to zero, (a) static waves appear under mass balance consumption, while (b) traveling waves emerge under constant resource assumption. Solid line represents the steady state and dotted lines represent waves that change over time.
(TIF)

S8 Fig Examples of simulated size spectra with a constant background resource. Different values of (a) the reproduction rate factor *c*, (b) the reproduction rate exponent *r*, (c) the metabolic rate exponent *q* with a small metabolic rate factor *v*, and (d) the exponent *q* with a large factor *v* are used for simulations. Solid lines represent size spectra obtained from running the system to steady state, while dotted lines represent collapsed communities that will never reach equilibrium.
(TIF)

S1 File Calculating the number of individuals *G*_*i*_ and *B*_*i*_. (DOCX)

S2 File Matlab code. (DOCX)
