# Supplementary figures and images for: A size-structured matrix model to simulate dynamics of marine community size spectrum
Source: PLoS One. 2018 Jun 7;13(6):e0198415. doi: 10.1371/journal.pone.0198415 (PMC5991710; doi:10.1371/journal.pone.0198415)

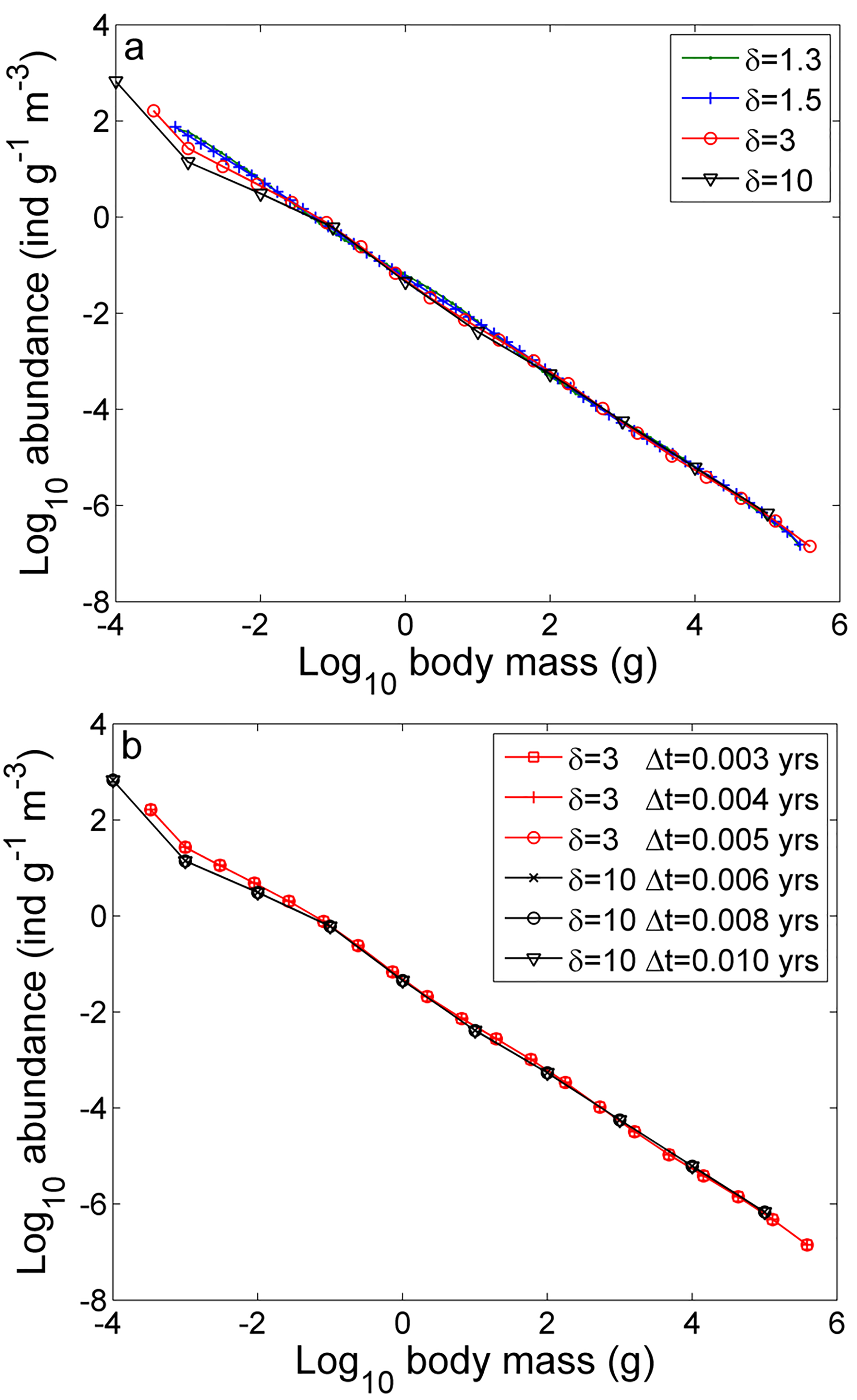

Supplement: S1 Fig — Normalized size spectra, in which the abundance is divided by the logarithmic width of each size class, at steady states for different values of (a) mass ratio δ of successive size classes and (b) time step Δt. (TIF) [file pone.0198415.s001.tif]

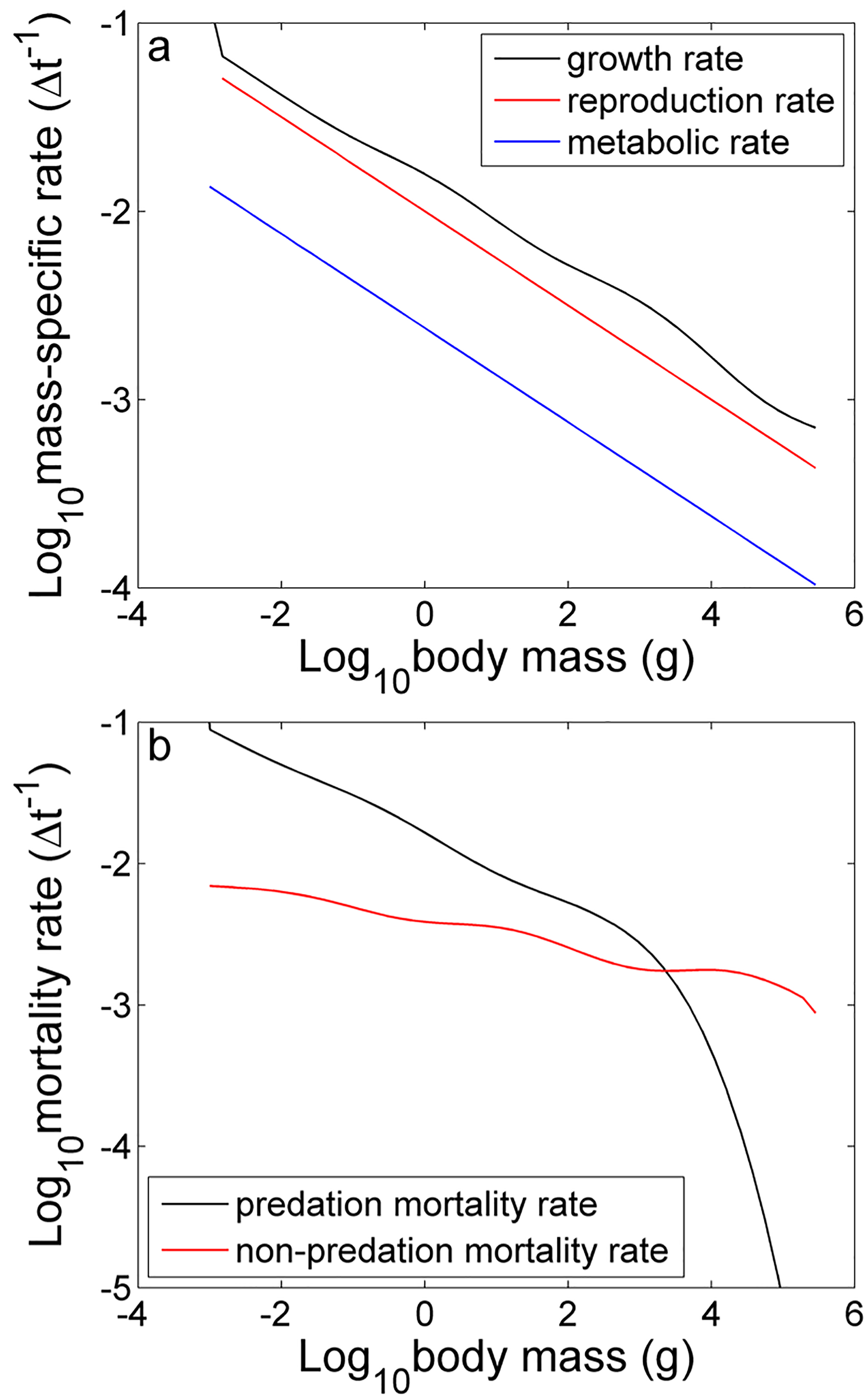

Supplement: S2 Fig — (a) Decrease in mass-specific growth, reproduction, and metabolic rates with increasing size. (b) Decrease in predation mortality, and non-predation mortality rates with increasing size. (TIF) [file pone.0198415.s002.tif]

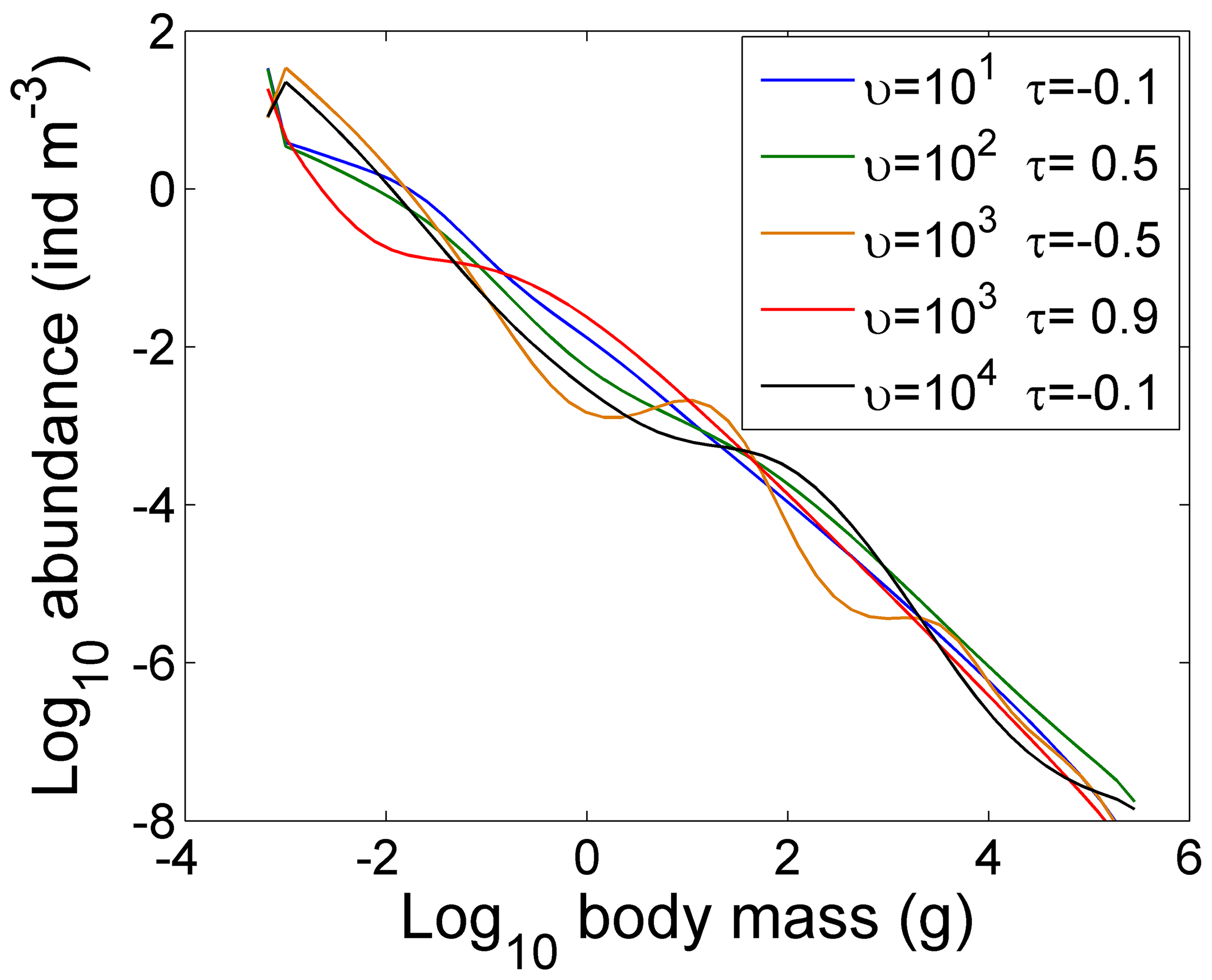

Supplement: S3 Fig — Simulated size spectra at steady state for different combinations of factor υ and exponent τ of PPMR. (TIF) [file pone.0198415.s003.tif]

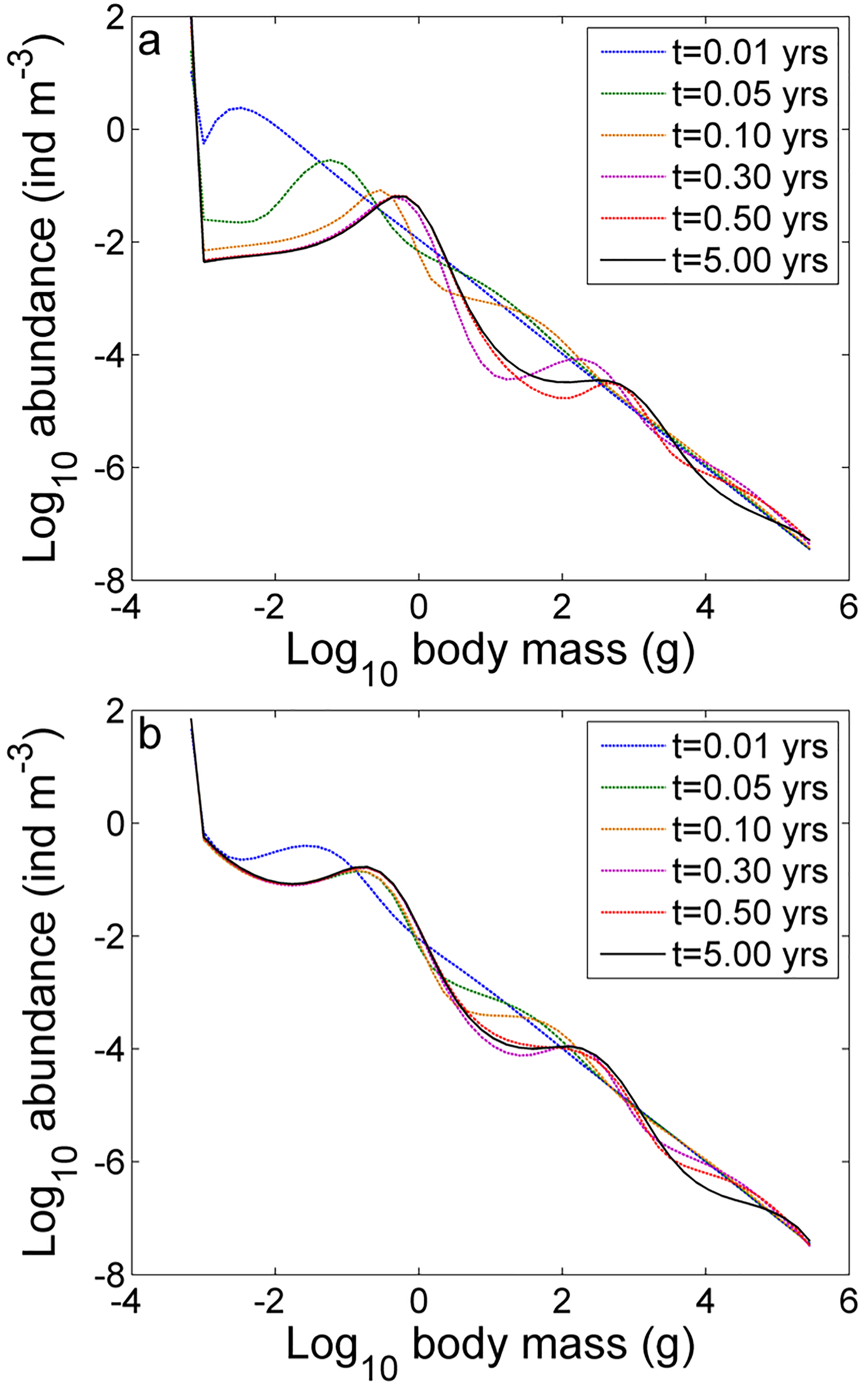

Supplement: S4 Fig — When (a) the reproduction rate factor c is set to 0.1 and (b) the metabolic rate factor v and exponent q are set to 5 and 0.25, respectively, the model produces transient oscillations until the system reaches a stationary state. Dotted lines represent the waves propagating through the spectrum, while solid lines represent the stationary states (i.e., static waves). (TIF) [file pone.0198415.s004.tif]

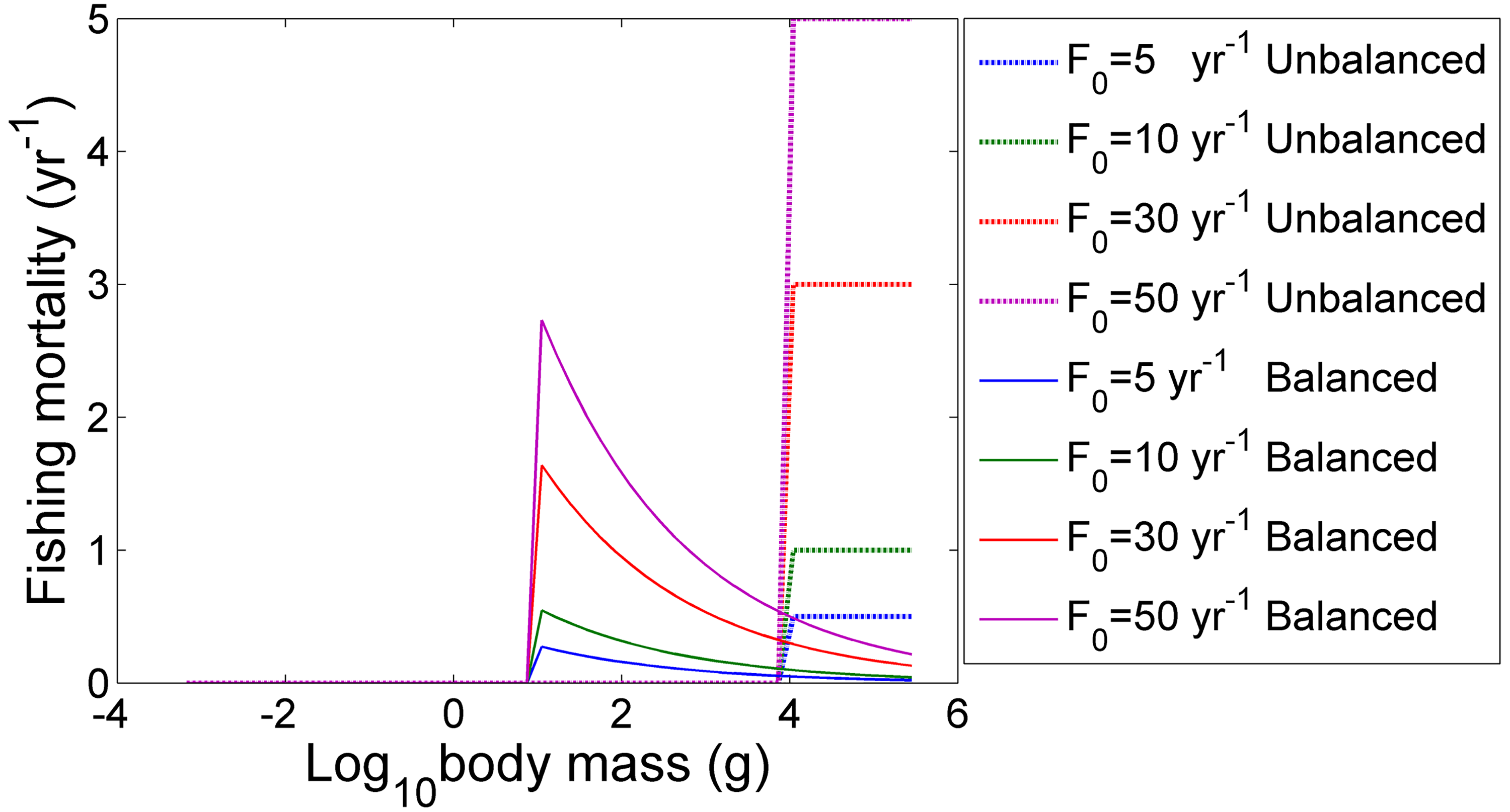

Supplement: S5 Fig — Relationship between body mass and fishing mortality for four different fishing intensity coefficients F0 under unbalanced and balanced fishing patterns. (TIF) [file pone.0198415.s005.tif]

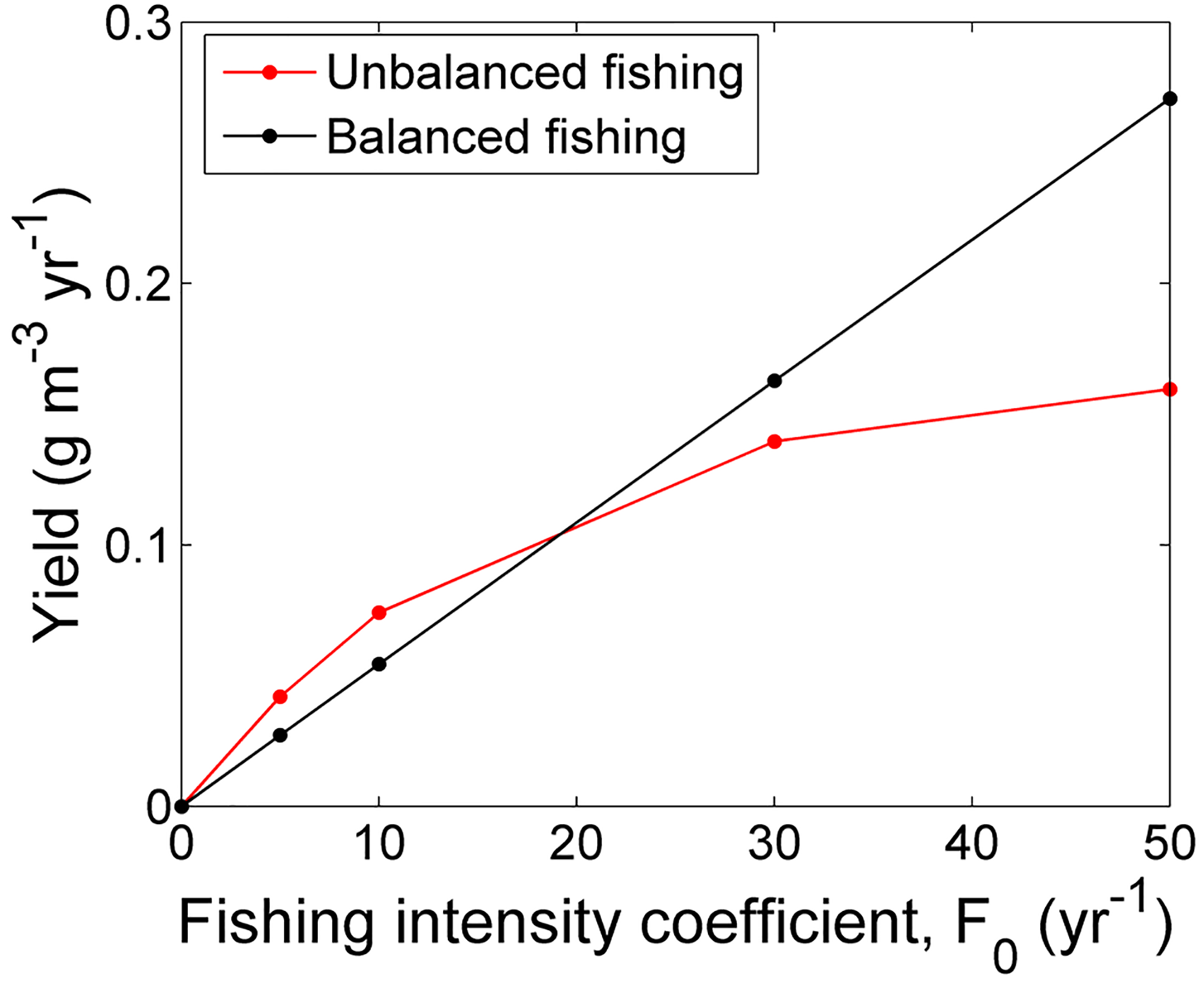

Supplement: S6 Fig — (TIF) [file pone.0198415.s006.tif]

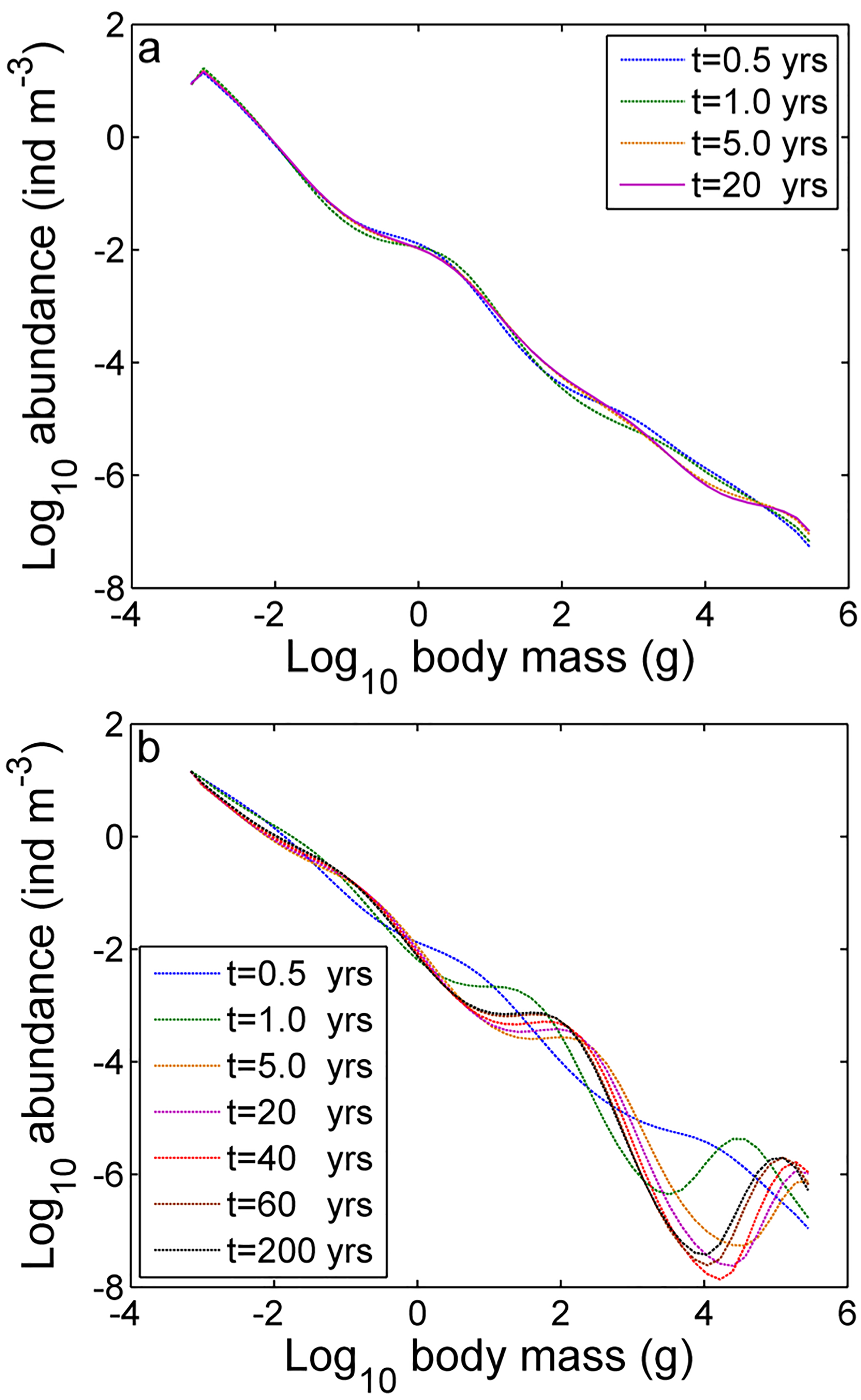

Supplement: S7 Fig — When the non-predation mortality rate factor μ0 is set to zero, (a) static waves appear under mass balance consumption, while (b) traveling waves emerge under constant resource assumption. Solid line represents the steady state and dotted lines represent waves that change over time. (TIF) [file pone.0198415.s007.tif]

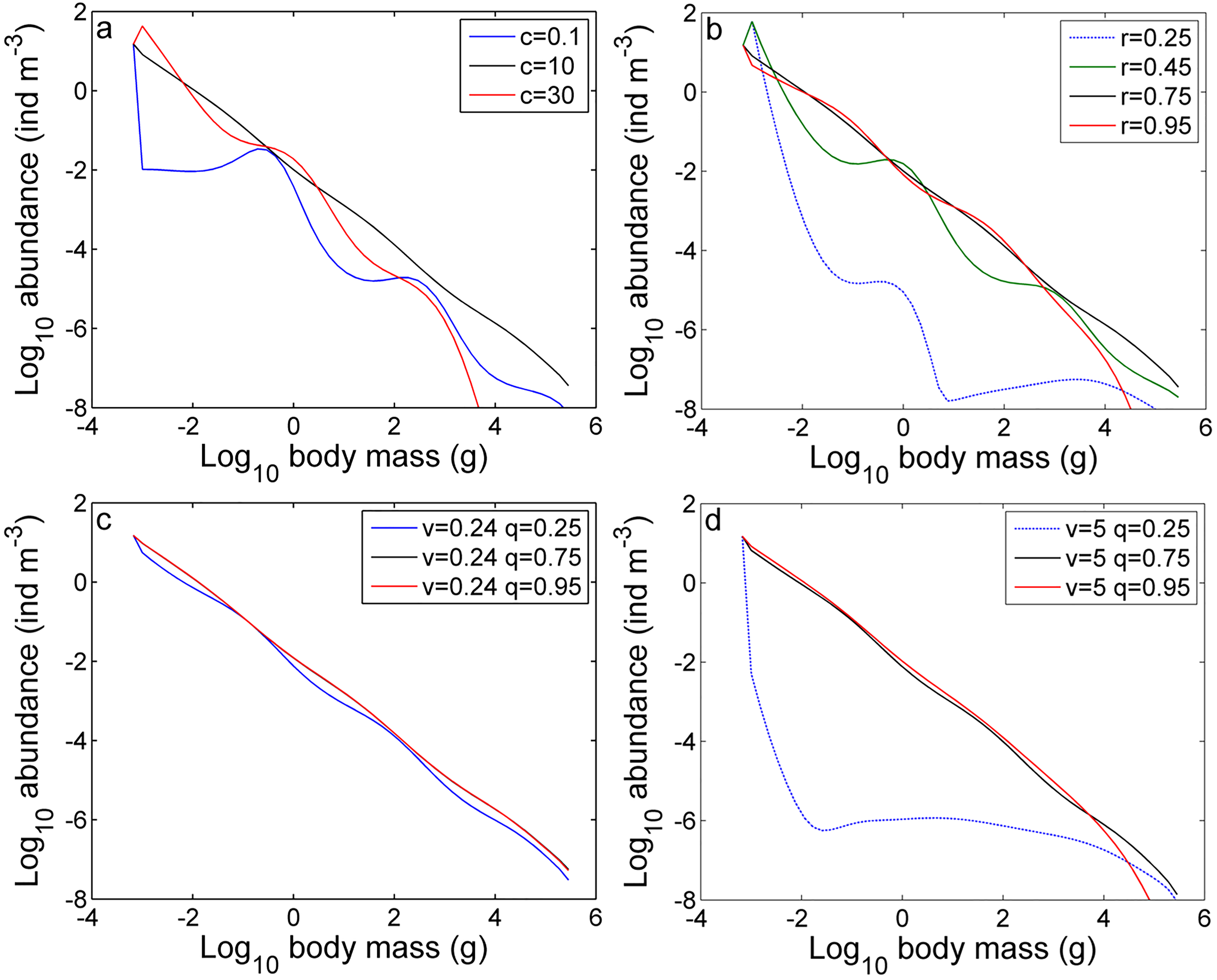

Supplement: S8 Fig — Different values of (a) the reproduction rate factor c, (b) the reproduction rate exponent r, (c) the metabolic rate exponent q with a small metabolic rate factor v, and (d) the exponent q with a large factor v are used for simulations. Solid lines represent size spectra obtained from running the system to steady state, while dotted lines represent collapsed communities that will never reach equilibrium. (TIF) [file pone.0198415.s008.tif]
