# Supplementary material for: A size-structured matrix model to simulate dynamics of marine community size spectrum
Source: PLoS One. 2018 Jun 7;13(6):e0198415. doi: 10.1371/journal.pone.0198415 (PMC5991710; doi:10.1371/journal.pone.0198415)
Supplement: S1 File — (DOCX) [file pone.0198415.s009.docx]

**S1 File**

The transitions of individuals from size class *i* to class *i*+1 as a result of growth and to class *i*-1 as a result of mass decrease in the processes of reproduction and metabolism can be calculated based on a discretization of the McKendrick-von Foerster equation using the following differencing scheme:

$$\frac{n_{i}^{t+\Delta t}-n_{i}^{t}}{\Delta t}=-\frac{g_{i}n_{i}^{t}-g_{i-1}n_{i-1}^{t}}{\Delta m_{i}}-\mu_{i}n_{i}^{t}, i>2$$

where *g_i_* is the assimilation rate, Δ*m_i_* is the mass difference between size classes *i*+1 and *i*, and *μ_i_* is the total mortality rate. The number of individuals *G_i_* in size class *i* growing to class *i*+1 is then represented as *g_i_n_i_* / (*m_i_*_+1_－*m_i_*). In a similar manner, the number of individuals *B_i_* in size class *i* moving to size class *i*-1 can be represented as *F_i_n_i_* / (*m_i_*－*m_i_*_-1_).
