# Supplementary material for: A size-structured matrix model to simulate dynamics of marine community size spectrum
Source: PLoS One. 2018 Jun 7;13(6):e0198415. doi: 10.1371/journal.pone.0198415 (PMC5991710; doi:10.1371/journal.pone.0198415)
Supplement: S2 File — (DOCX) [file pone.0198415.s010.docx]

**S2 File**

N=50;% total number of size classes

i=1:N;

j=1:N;

delta=1.5;% mass ratio of successive size classes

t=0.001;% time step

% body mass axis

m=ones(N,1);

m(i)=0.001*delta.^(i-2);

% initial number of individuals in each size class

n=0.01*m.^(-1);

% generate transition matrix T1

T1=zeros(N,N);

T1(1,j)=1;

% generate transition matrix T2

T2=zeros(N,N);

T2(2,j)=1;

upsilon=100; % the factor of PPMR

tau=0; % the exponent of PPMR

sigma=1.3; % width of size preference

Ns=round(7/log10(delta));% the total number of resources' size classes

% generate size selectivity matrix S

s=1:Ns;

x=ones(Ns,1);

x(s)=0.001*delta.^(-Ns+s-1);

X=[x;ones(N-Ns,1)];

S=exp(-(log(upsilon*(repmat(m',N,1))'.^tau.*repmat(m',N,1)...

./(repmat(m',N,1))')).^2/2/sigma^2);

Ss=exp(-(log(upsilon*(repmat(m',N,1))'.^tau.*repmat(X',N,1)...

./(repmat(m',N,1))')).^2/2/sigma^2);

Y=sum(Ss(:,1:Ns),2);

S(:,1)=Y;

S=tril(ones(N,N),-1).*S;

gamma=600;% the factor of searching rate

p=0.75;% the exponent of searching rate

V=diag(gamma*m.^p);% searching rate matrix V

Tg=diag(ones(N-1,1),-1)-eye(N);% transition matrix Tg

% generate transition matrix Tc

Tc=zeros(N,N);

Tc(1,N)=delta*m(N)/m(1);

% generate the mass difference matrix ΔM+

Mp=diag([m(2:end)-m(1:end-1);0]);

Mp(N,N)=delta*m(N)-m(N);

% generate the mass difference matrix ΔM-

Mm=diag([0;m(2:end)-m(1:end-1)]);

Mm(1,1)=m(1);

e=0.6;% assimilation efficiency

E=e*eye(N);% assimilation efficiency matrix E

z0=400;% the factor of non-predation mortality rate

z=-0.9;% the exponent of non-predation mortality rate

% generate non-predation mortality rate matrix D

D=diag(z0*m.^(-z));

D(1,1)=0;

M1=m(1)*eye(N);% matrix M1

I=eye(N);% unit matrix I

alpha=0.5;% sex ratio

A=alpha*eye(N);% sex ratio matrix A

c=10;% the factor of reproduction rate

r=0.75;% the exponent of reproduction rate

% generate reproduction rate matrix R

R=diag(c*m.^r);

R(1,1)=0;

R(2,2)=0;

v=2.4;% the factor of standard metabolic rate

q=0.75;% the exponent of standard metabolic rate

% generate metabolic rate matrix Q

Q=diag(v*m.^q);

Q(1,1)=0;

M2=m(2)*eye(N);% matrix M2

H=zeros(N,N);% fishing mortality rate matrix H

iteration=20000;

figure

L1=plot(log10(m),log10(n),'r');

legend(L1,'initial condition');

xlabel('Log_{10} body mass (g)');

ylabel('Log_{10} abundance (ind m^{-3})');

hold on

count = 0;

disp('Simulation in process, please wait...');

while count < iteration

drawnow

count = count + 1;

temp = n;

n = n-(diag(n)*S'*V-(Tg+Tc)*inv(Mp)*E*diag(n)*V*S*diag(m)...

+diag(n)*D+H-T1*inv(M1)*((I-E)* diag(n)*V*S* diag(m)+(I-A)*R...

+Q+(diag(n)*D+H)*diag(m))-T2*inv(M2)*A*R-Tg'*inv(Mm)*(R+Q))*n*t;

if temp == n

L2=plot(log10(m),log10(n),'ko-');

legend(L2,'stationary condition');

disp('equilibrium:');

disp(n');

disp('iteration:');

disp(count);

break;

end;

if mod(count,1000) == 0

plot(log10(m),log10(n),'b');

end

end

hold off

disp(n);

disp('Finished');
